# Chronic administration of fluoxetine and pro-inflammatory cytokine change in a rat model of depression

**DOI:** 10.1371/journal.pone.0186700

**Published:** 2017-10-19

**Authors:** Yanxia Lu, Cyrus S. Ho, Xin Liu, Anna N. Chua, Wei Wang, Roger S. McIntyre, Roger C. Ho

**Affiliations:** 1 Department of Clinical Psychology and Psychiatry/School of Public Health, Zhejiang University College of Medicine, Hangzhou, China; 2 Department of Psychological Medicine, Yong Loo Lin School of Medicine, National University of Singapore, Singapore; 3 Department of Psychological Medicine, National University Health System, Singapore; 4 Shandong Provincial Key Laboratory of Cerebral Microcirculation, Taishan Medical University, Tai’an, China; 5 Department of Medical Psychology, School of Basic Medical Sciences, Taishan Medical University, Tai’an, China; 6 Institute of Medical Science, University of Toronto, Toronto, Ontario, Canada; 7 Mood Disorders Psychopharmacology Unit, University Health Network, Toronto, Ontario, Canada; 8 Department of Psychiatry, University of Toronto, Toronto, Ontario, Canada; 9 Department of Toxicology and Pharmacology, University of Toronto, Toronto, Ontario, Canada; Radboud University Medical Centre, NETHERLANDS

## Abstract

This study evaluated the chronic effects of fluoxetine, a commonly prescribed SSRI antidepressant, on the peripheral and central levels of inflammatory cytokines including IL-1β, IL-6, TNF-α and IL-17 over a 4-interval in a rat model of chronic mild stress (CMS) which resembles the human experience of depression. Twenty-four Sprague-Dawley rats were randomly assigned to CMS+vehicle (n = 9), CMS+fluoxetine (n = 9) and the control (n = 6) groups. Sucrose preference and forced swim tests were performed to assess behavioral change. Blood samples were collected on day 0, 60, 90 and 120 for measurement of cytokine levels in plasma. On day 120, the brain was harvested and central level of cytokines was tested using Luminex. Four months of fluoxetine treatment resulted in changes in the sucrose preference and immobility time measurements, commensurate with antidepressant effects. The CMS+vehicle group exhibited elevated plasma levels of IL-1β, IL-17, and TNF-α on day 60 or 120. Rats treated with fluoxetine demonstrated lower IL-1β in plasma and brain after 90 and 120-day treatment respectively (*p*<0.05). There was a trend of reduction of IL-6 and TNF-α concentration. This study revealed the potential therapeutic effects of fluoxetine by reducing central and peripheral levels of IL-1β in the alleviation of depressive symptoms.

## Introduction

Studies suggest that depression is a common, debilitating mood disorder that affects approximately 20% of the global population [1, 2]. Depression is not only the third leading cause of disease burden globally but also exerts a significant socioeconomic burden worldwide [3, 4]. The burden of illness associated with depression, as well as insufficient outcomes with current treatments, invites the need for a more refined understanding of the disease state.

Recent studies have provided compelling evidence that inflammation plays a pivotal role in subserving depression symptom phenomenology with observations that (i) patients with interferon (IFN)-α or interleukin (IL)-2 therapy developed depressive symptoms; (ii) endotoxin lipopolysaccharide (LPS) induced the secretion of multiple inflammatory cytokines which in turn cause typical depressive symptoms such as sickness behaviour; (iii) dysregulation of the hypothalamic–pituitary–adrenal (HPA) axis activity, commonly observed in depressed patients, can be induced by several cytokines; (iv) some cytokines are involved in the regulation of brain norepinephrine or serotonin systems, which is associated with major depression disorder and its treatment [5, 6].

Animals studies suggest that external and internal stress can activate innate inflammatory immune response and alter the ability of immune cells to secrete inflammatory cytokines, resulting in sickness behaviors (e.g. changes in psychomotor activity, food intake, and social interaction) and anhedonia [7]. Stress is associated with increased plasma/serum and cerebrospinal fluid concentration of inflammatory cytokines (e.g., TNF-α, IL-1β, and IL-6) in depressed patients compared with healthy subjects [8–12]. Evidence also indicates that a relationship exists between measures of anhedonia and alterations in brain structure/function. For example, greater regional cerebral blood flow was observed in the left brain hemisphere when healthy subjects have presented a meal after a prolonged fast [13, 14]. A study by Porubska *et al*. (2006) [15] reported increase metabolism primarily and most markedly in the left hemisphere when appetite was induced in healthy subjects by viewing food pictures. However, existing findings are mainly based on results derived from cross-sectional studies or those with a relatively short follow-up duration; the chronic impact of antidepressants on the peripheral and central levels of inflammatory cytokines in treating depression has rarely been investigated.

The selective serotonin reuptake inhibitors (SSRIs) such as fluoxetine [16] are currently adopted as first-line treatments for depression because of their superior safety and tolerability profiles compared with other antidepressants (e.g. tricyclic antidepressants (TCAs) and monoamine oxidase inhibitors (MAOIs)) [17–19]. It is hypothesized that SSRIs could effectively reduce left brain hemisphere activity [20] and lower the levels of pro-inflammatory cytokines [21] in depressed patients. The limitation of technology in measuring cytokine levels directly from the brain of depressed patients leads to the necessity of animal model. This study aimed to dynamically evaluate the impact of fluoxetine on altering the peripheral (plasma) and central (left brain hemisphere) levels of inflammatory cytokines including IL-1β, IL-6, TNF-α and IL-17 at baseline, day 60, day 90 and day 120 in alleviating depression in a 4-month (equal to 12 years of human [22]) rat model of chronic mild stress (CMS) [23, 24] which closely mirrors depression that manifested in humans after daily stressful life events as opposed to traumatic events introduced in acute stress models [25, 26].

## Materials and methods

### Animals

The study was approved by the Institutional Animal Care and Use Committee (IACUC) of National University of Singapore. Twenty-four 6–8 week-old female adult Sprague-Dawley rats, weighing 182–292 grams, were used in this experiment. Mimicking gender difference in stress response and depression in humans, female SD rats are more susceptible to the behavioural, endocrine, and molecular response of the stress systems, and are thus used for depression models [27]. Animals were housed individually in a ventilated cage with free access to pelleted rodent diet and water *ad libitum*. The holding room was maintained at room temperature of 22–25°C with a humidity of 55±10% and 12-hour light/day cycle. Rats were allowed to habituate to the new surrounding for three days and baseline behavioral data was recorded prior to any treatments for water and sucrose preference, as described in section 2.3. All procedures were performed in accordance with institutional guidelines with every effort made to minimize the suffering of animals.

### Outline of study

The animals were randomly divided into 3 groups: the control group, the chronic mild stress (CMS)+vehicle group and the CMS+fluoxetine group. Rats in the control group (n = 6) were not subjected to any stress and left undisturbed in their room under maintenance condition. The other 2 groups were subjected to chronic mild stress procedure daily for 4 months. The CMS+fluoxetine group received additionally chronic fluoxetine treatment (a gavage of 0.042mg/g once daily dissolved in 0.5ml distilled water per rat), and the CMS+vehicle group received a gavage of 0.5ml of distilled water once daily. Both groups were administered the solution by gavage on a daily basis at the similar time of the day. Blood samples were collected from all rats via tail vein into centrifuge tubes pre-treated with ethylenediaminetetraacetic acid (EDTA) at baseline, and on day 60, day 90 and day 120. Blood samples were centrifuged at 4°C, 1000 g for 10mins to isolate serum. On day 120, rats were anesthetized with sodium pentobarbital and their brains were harvested on the ice. The left hemisphere was homogenized in a radio-immunoprecipitation assay (RIPA) buffer composed of Tris-HCl (pH 7.4), 5M NaCl, 0.5M EDTA, Na_3_VO_4_ and 0.2ml NaF. The obtained homogenate was subject to shaking at 4°C for 2 hours followed by centrifuge. After that, the supernatants were aliquoted and stored at -80°C until further analysis.

### Behavioural tests

#### Chronic mild stress (CMS) procedure

The CMS procedure in this study lasted for 4 months and the protocol was reported by Willner et al. (1987) [28] with modifications based on previous research [29] and recommendations from the local ethics committee. Each rat from the CMS+vehicle group and the CMS+fluoxetine group was subject to continuous single housing and one stressor per day based on a fixed weekly schedule. The stressors were: (1) food deprivation for 18 h; (2) continuous overnight illumination; (3) soiled cage with 100 ml of water spilled onto the bedding for 6h; (4) cold water swimming at 18°C for 5 mins; (5) shaking on a rocking bed at orbital motion of 200 rpm for 15 mins; (6) physical restraint for 20 mins; and (7) water deprivation for 18 h. The body weight of each rat was measured on a weekly basis for calculation of mean body weight changes during CMS period.

#### Sucrose preference test

The sucrose preference test was executed to operationally define anhedonia [28–30]. Each rat was subjected to three 1-h training session before any CMS or fluoxetine treatment and baseline of sucrose preference was recorded in the final training session. The test was conducted under a similar condition at the end of 4-month and results were analyzed against a baseline level of sucrose preference intake. The training consisted of making a free choice of either drinking 1% (w/v) sucrose solution or distilled water presented to them succeeding 23-h food and water deprivation [28, 31]. At the end of each test, distilled water and sucrose intake were calculated by measuring the differences in weights of respective bottles prior to and after consumption. The percentage of sucrose preference (SP) was calculated in accordance with the following formula: SP% = sucrose intake/(sucrose intake+water intake)*100.

#### Forced swim test (FST)

The FST is a standard test to determine the state of depression in rats. The test was implemented based on the protocol reported by Porsolt et al. (1978) with slight modifications to better assess the effects of an antidepressant [32] at the end of the CMS procedure on day 120. A 15-minute training session was conducted 24 h before the FST. During the FST, rats were dropped individually into a vertical plexiglass cylinder (height: 30 cm, diameter: 22.5 cm) filled with water (23–25°C) to a depth of 40cm. The test lasted for 2 mins per rat because the effects of antidepressants on immobility are suggested better distinguished from saline when parameters were scored in the first 2 min [33], and duration of immobility was recorded once the rat acquired an immobile posture upon initial vigorous activity.

### Measurement of peripheral and central levels of inflammatory cytokines

Levels of IL-6, IL-1β, IL-17, and TNF-α were quantified in plasma isolated from blood samples collected at baseline and on day 60, 90 and 120, as well as in supernatants of left brain hemisphere homogenate obtained on day 120. Experiments were performed following the instructions of the commercially available multiplex bead-based immunoassay (EMD Millipore, Catalogue RECYTMAG-65K). All samples were assayed in duplicate. Briefly, plasma and brain samples were thawed to 4°C and centrifuged for 3 mins at 4°C, 1000g to settle any residue. The immunoassay was performed with beads coated with anti-IL-6, anti-IL-17, anti-IL-1β and anti-TNF-α antibodies that were added after the addition of 25μl of standards and samples into respective wells of microtiter filter plates. This was followed by 2 h incubation at room temperature with shaking at 450 rpm on the plate shaker. The plate was then washed and incubated for 1 h at room temperature with detection antibodies followed by another 30-min incubation with the addition of streptavidin-phycoerythrin. Then, the plate was washed twice and sheath fluids were added to all wells. Subsequently, the plate was read with Luminex 200 plate reader and standard curves of four different cytokines (IL-6, IL-1β, IL-17 and TNF-α) ranging from 2.4–73.2pg/ml up to 10,000–300,000 pg/ml were automatically constructed via the five-parameter logistic method. The cytokine concentrations of experimental samples were calculated based on the standard curves generated.

### Statistical analysis

Statistical analysis was performed based on the distribution of data regarding respective parameters. Results generated were expressed as a mean±standard error (S.E.M). The effect of fluoxetine on FST score and the levels of inflammatory cytokines (IL-1β, IL-6, IL-17, and TNF-α) was determined by one-way analysis of variance (ANOVA) for normally distributed data, or Kruskal-Wallis test for non-normally distributed data. The mean differences among the CMS+fluoxetine, the CMS+vehicle, and the control groups were considered significant if *p*<0.05. If statistical significance is detected by one-way ANOVA, post-hoc pairwise comparison was further performed using Tukey analysis when homogeneity of variance was satisfied (*p*>0.05) or Games-Howell analysis when homogeneity of variance was violated (*p*<0.05). If statistical significance is achieved in Kruskal-Wallis test, multiple Mann-Whitney U tests were further conducted for pairwise comparison. Furthermore, Wilcoxon signed-rank test was performed to compare the levels of cytokines in plasma at baseline versus each time point of blood collection during follow-up (day 60, day 90 and day 120). The effect of fluoxetine on sucrose preference and body weight on day 120 was analyzed by paired-sample t-test. All analyses were conducted using Statistical Package for the Social Sciences (SPSS, version 21.0).

## Results

**Fig 1:**
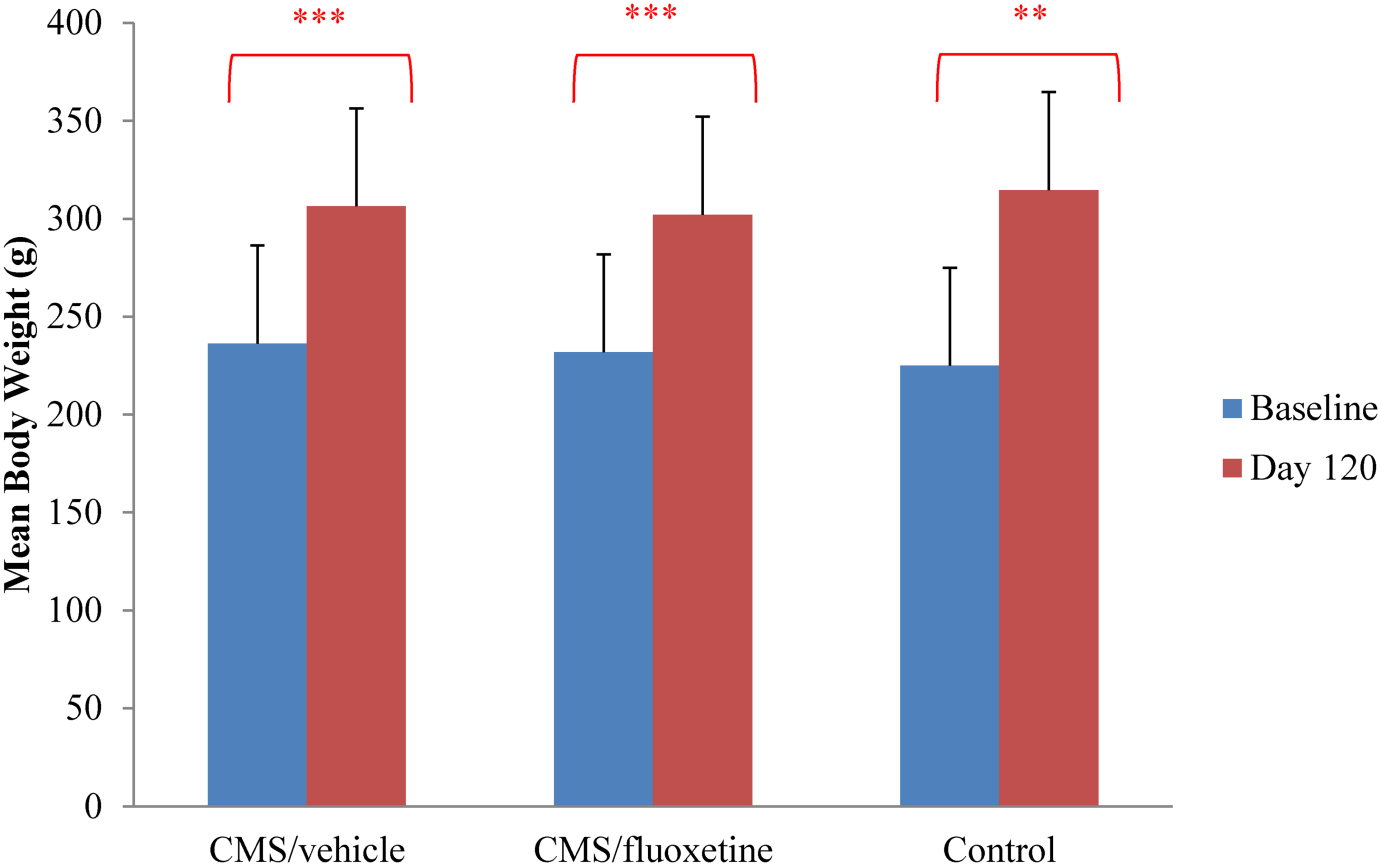
Change of mean body weight from baseline to day 120. Data are presented in the CMS+fluoxetine (0.042mg/g once daily dissolved in 0.5ml distilled water per rat) group (n = 9), the CMS+vehicle (0.5ml distilled water per rat once daily) group (n = 9) and the control group (n = 6) respectively. Paired-sample t-test was used for the statistical analysis. ***p<0.001, **p<0.01 *vs*. baseline. CMS = chronic mild stress.

The change of body weight is shown in Fig 1 on day 120 as compared to the baseline. Significant increase was observed in mean body weight for the CMS+fluoxetine (0.042mg/g once daily dissolved in 0.5ml distilled water per rat) group (t(8) = 8.425, df = 8, *p*<0.001), the CMS+vehicle group (t(8) = 6.636, df = 8, *p*<0.001) and the control group (t(5) = 6.782, df = 5, *p* = 0.001). Specifically, the control group showed the greatest mean weight gain of 89.7 g, with baseline and day 120 mean body weight of (225.0±13.1) g and (314.7±28.1) g respectively. The CMS+vehicle (0.5ml distilled water per rat once daily) group demonstrated an average weight gain of 70.1 g, with baseline and day 120 mean body weight of (236.3±8.6) g and (306.4±7.9) g respectively. As for the CMS+fluoxetine group, rats gained 70.2 g weight during the 4-month experiment, with baseline and day 120 mean body weight of (231.9±5.7) g and (302.1±8.9) g respectively.

**Fig 2:**
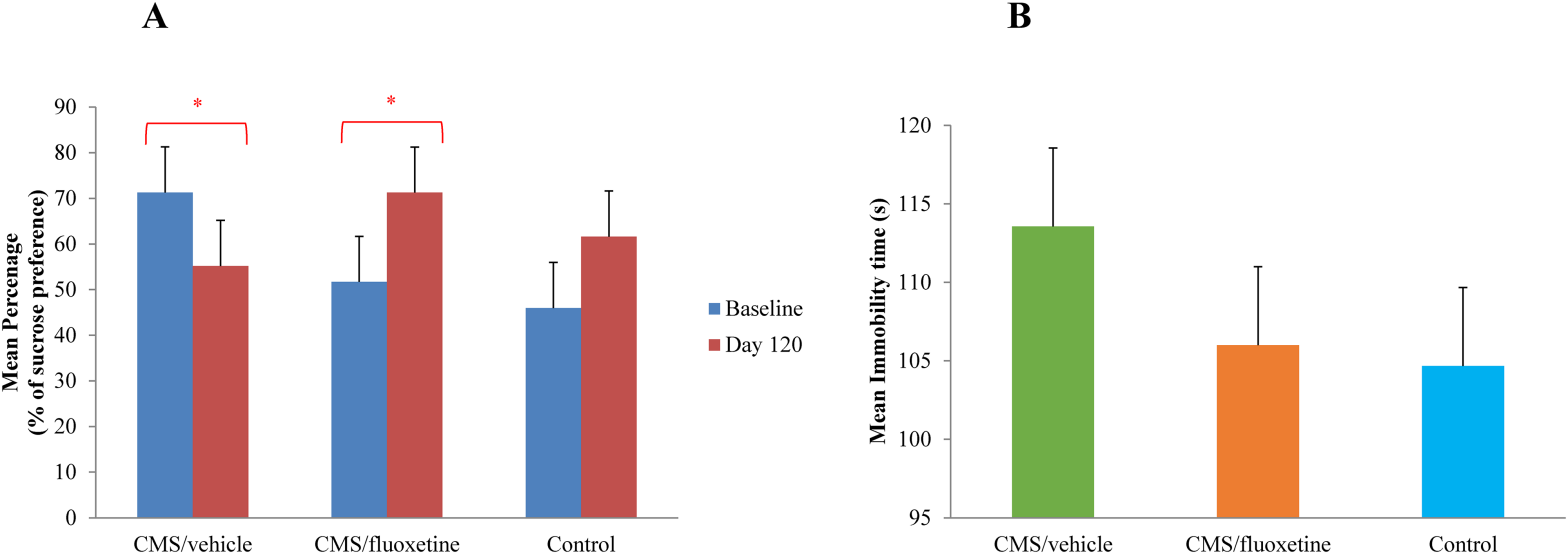
Comparison of time and group difference of behavioral test scores. A) Mean percentage of sucrose preference at baseline and on day120 in the CMS+fluoxetine group (n = 9), CMS+vehicle group (n = 9) and control group (n = 6) by paired-sample t-test. *p<0.05 *vs*. baseline. B) The effect of FST for CMS+fluoxetine group, CMS+vehicle group and control in the mean immobility time recorded on day 120 by one-way ANOVA. Bars represent a mean ± standard error. CMS = chronic mild stress; FST = force swim test; ANOVA = analysis of variance.

Fig 2A demonstrated the mean percentage of sucrose preference in different study groups recorded on day 120 as compared to the baseline level. Paired-sample t-test revealed a significant decrease in the mean percentage of sucrose preference in the CMS+vehicle group (t(8) = -2.592, df = 8, *p* = 0.032) from baseline (71.3±3.6)% to day 120 (55.2±5.1)%, suggesting the development of anhedonia in the CMS+vehicle group and the success of the CMS model in the present study. There was a significant increase in the mean percentage of sucrose preference in the CMS+fluoxetine (t(8) = 2.686, df = 8, *p* = 0.028) on day 120 (71.2±6.1)% as compared to baseline (51.7±4.8)%. However, the control group (t(5) = 1.023, df = 5, *p* = 0.353) exhibited no significant change in the mean percentage of sucrose preference over the 4-month period. Mean immobility time was recorded on day 120 during the FST and is shown in Fig 2B. One-way ANOVA revealed no significant difference (F (2,21) = 0.069, df = 2, *p* = 0.934) in the mean immobility time among the CMS+fluoxetine, the CMS+vehicle, and the control groups. Nevertheless, there was a trend of lower mean immobility time in the CMS+fluoxetine (106.00±20.35) s and control (104.67±14.86) s groups as compared to the CMS+vehicle group (113.56±16.96) s.

Table 1 shows the levels of four inflammatory cytokines (IL-1β, IL-17, IL-6, and TNF-α) in the plasma of rats for the CMS+fluoxetine, the CMS+vehicle and the control groups, from day 0 to day 120 by one-way ANOVA (for normally distributed data) and Kruskal-Wallis test (for non-normally distributed data). These analyses were conducted with respect to the normality of distribution in each group. One-way ANOVA revealed significant differences in the plasma levels of IL-1β on day 90 (F = 5.463, df = 2, *p* = 0.026), but no significant difference in the plasma levels of IL-1β on day 60 (F = 1.480, df = 2, *p* = 0.250) among the CMS+fluoxetine, the CMS+vehicle, and the control groups. Games-Howell post hoc analysis revealed that the levels of IL-1β in plasma was significantly lower (*p*<0.01) in the CMS+fluoxetine group (187.78±53.28) pg/ml than in the CMS+vehicle group (676.42±136.94) pg/ml on day 90. There was a trend of lower IL-1β levels in the control group (403.16±185.91) pg/ml compared with the CMS+vehicle group (676.42±136.94) pg/ml on day 90, but the difference did not achieve statistical significance (*p*>0.05). There was no significant difference in the plasma levels of IL-1β on day 0 (χ^2^ = 0.504, df = 2, *p* = 0.777) or day 120 (χ^2^ = 5.400, df = 2, *p* = 0.067) among the three study groups by Kruskal-Wallis test. However, Kruskal-Wallis test revealed no significant difference (*p*>0.05) in the plasma levels of IL-6, TNF-α or IL-17 from day 0 to day 120.

**Table 1:** Comparison of mean plasma levels of inflammatory cytokines from day 0 to day 120.

Cytokines	Time	Groups			Test statistical value
		CMS+fluoxetine (n = 9)	CMS+vehicle (n = 9)	Control (n = 6)	
**IL-1β**	Day 0 (mean±SEM)^#^	344.31±114.33	326.65±125.75	357.35±73.45	χ² = 0.504, df = 2, *p* = 0.777
	Day 60 (mean±SEM)	206.06±78.41	492.09±155.78	364.56±119.61	F = 1.480, df = 2, *p* = 0.250
	Day 90 (mean±SEM)	187.78±53.28**	676.42±136.94	403.16±185.91	F = 5.463, df = 2, *p* = 0.026
	Day120 (mean±SEM) ^#^	141.05±56.00	976.56±360.92	390.65±197.24	χ² = 5.400, df = 2, *p* = 0.067
**IL-6**	Day 0 (mean±SEM) ^#^	34.23±34.23	0.00±0.00	74.85±47.34	χ² = 3.205, df = 2, *p* = 0.201
	Day 60 (mean±SEM) ^#^	1.49±1.49	7.87±7.87	0.00±0.00	χ² = 0.700, df = 2, *p* = 0.705
	Day 90 (mean±SEM) ^#^	11.34±11.34	121.39±91.56	0.00±0.00	χ² = 3.164, df = 2, *p* = 0.206
	Day120 (mean±SEM) ^#^	17.32±17.32	1327.41±1108.59	0.00±0.00	χ² = 1.749, df = 2, *p* = 0.417
**TNF-α**	Day 0 (mean±SEM) ^#^	7.85±2.92	16.59±9.41	19.09±8.57	χ² = 0.428, df = 2, *p* = 0.807
	Day 60 (mean±SEM) ^#^	6.73±2.48	17.26±12.26	5.07±5.07	χ² = 4.593, df = 2, *p* = 0.101
	Day 90 (mean±SEM) ^#^	5.83±2.19	20.19±8.65	1.80±1.80	χ² = 3.221, df = 2, *p* = 0.200
	Day120 (mean±SEM) ^#^	14.17±5.30	51.85±22.94	11.77±11.77	χ² = 5.329, df = 2, *p* = 0.070
**IL-17**	Day 0 (mean±SEM) ^#^	5.56±3.45	6.67±4.80	4.15±4.15	χ² = 0.340, df = 2, *p* = 0.844
	Day 60 (mean±SEM) ^#^	6.05±2.29	6.45±6.45	4.05±4.05	χ² = 1.494, df = 2, *p* = 0.474
	Day 90 (mean±SEM) ^#^	5.15±2.17	14.3±8.98	5.10±3.40	χ² = 0.294, df = 2, *p* = 0.863
	Day120 (mean±SEM) ^#^	8.80±2.83	19.07±8.99	4.58±3.54	χ² = 1.992, df = 2, *p* = 0.369

CMS = chronic mild stress; IL = interleukin; SEM = standard error; TNF = tumour necrosis factor.

Data analysis was performed using one-way analysis of variance (ANOVA) and

^#^Kruskal-Wallis test.

**p<0.01 versus the CMS+vehicle group in Games-Howell post hoc test following one-way ANOVA.

**Fig 3:**
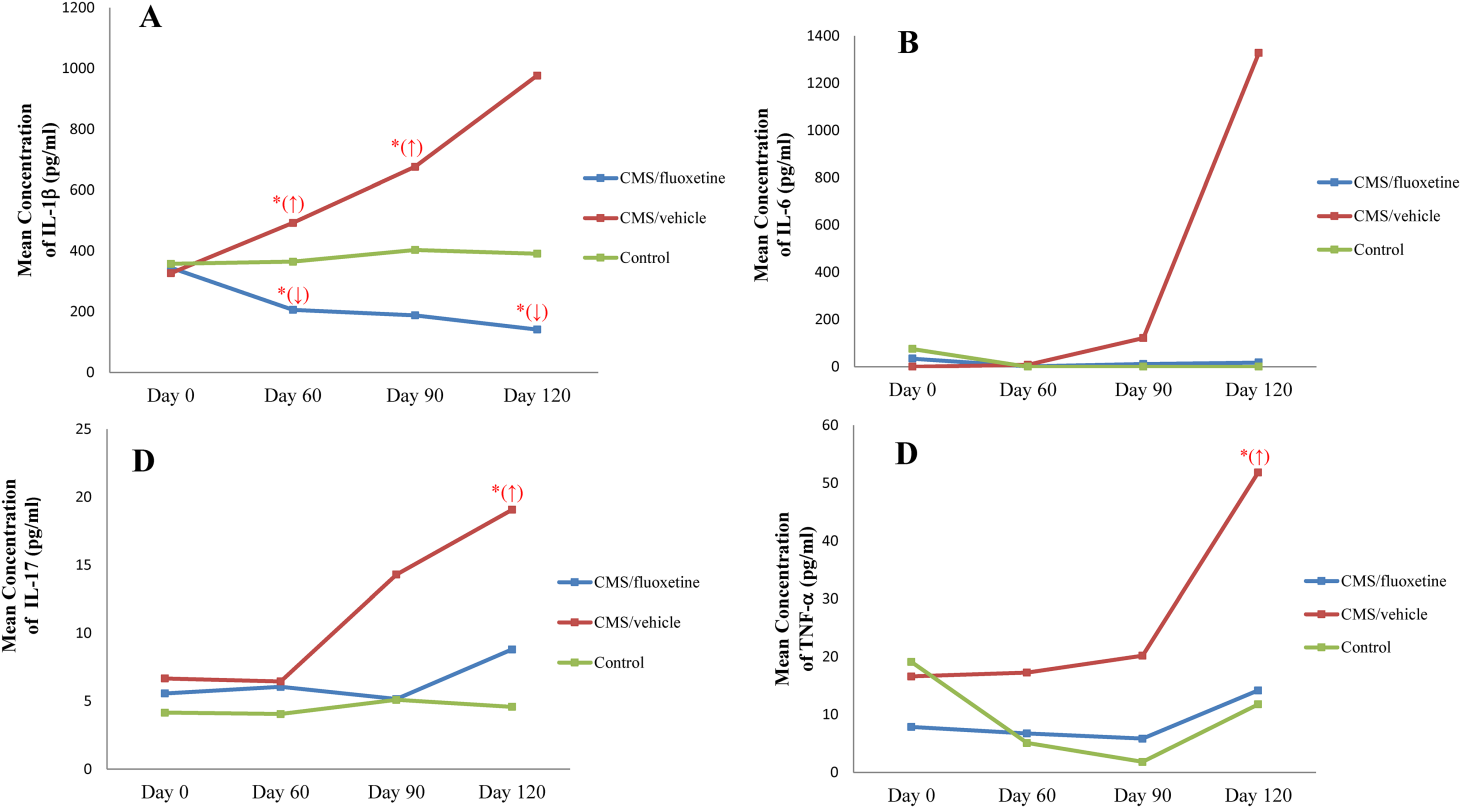
Change of mean concentration of inflammatory cytokines in plasma from baseline to day 60, day 90, and day 120. A) IL-1β; B) IL-6; C) IL-17; D) TNF-α. Data are presented in the CMS+fluoxetine group (n = 9), CMS+vehicle group (n = 9) and control group (n = 6). Wilcoxon signed-rank test was performed to investigate the time effect. *p<0.05 *vs*. baseline.

The change of inflammatory cytokine level in plasma at different time points against the baseline level is described in Fig 3. The CMS+fluoxetine group showed a trend of decrease in the plasma level of IL-1β from day 0 to day 120, with significant decrease in IL-1β level on day (60 (206.06±78.41) pg/ml, *p*<0.05) and day 120 ((141.05±56) pg/ml, *p*<0.05) as compared to its baseline level on day 0 (344.31±114.33) pg/ml via Wilcoxon signed-rank test. As for the CMS+vehicle group, there was a trend of increase in the level of IL-1β from day 0 to day 120; significant increase in IL-1β levels was demonstrated on day 60 ((492.09±155.78) pg/ml, *p*<0.05) and day 90 ((676.42±136.94) pg/ml, *p*<0.05) as compared to its baseline level on day 0 (326.65±125.75) pg/ml. The control group did not show any statistical significance in the plasma level of IL-1β over the 4-month period (*p*>0.05) (Fig 3A). Fig 3B shows the time effect of mean IL-6 levels (± SEM) in the plasma of the CMS+fluoxetine, the CMS+vehicle and the control groups at different time points against the baseline level. No statistical significance was detected in plasma IL-6 levels within the CMS+fluoxetine, the CMS+vehicle or the control groups over the 4-month period (*p*>0.05). Nevertheless, the CMS+vehicle group showed a trend of increase in the level of IL-6 from day 0 to day 120, while the CMS+fluoxetine group and the control group demonstrated a trend of lower IL-6 level on day 60 to day 120 as compared to the baseline level. As shown in Fig 3C, the CMS+vehicle group showed a trend of increase in the level of IL-17, with a significant increase on day 120 (19.07±8.99) pg/ml as compared to its baseline level (6.67±4.80) pg/ml via Wilcoxon signed rank test (*p*<0.05). However, the fluctuation of IL-17 level in plasma did not show statistical significance in the CMS+fluoxetine group and the control group over the 4-month period (*p*>0.05). Fig 3D demonstrated that the CMS+vehicle group had a trend of increase in the level of TNF-α, with a significant increase in TNF-α level on day 120 (51.85±22.94) pg/ml as compared to its baseline level on day 0 (16.59±9.41) pg/ml via Wilcoxon signed-rank test (*p*<0.05). No significant change was detected regarding the plasma level of TNF-α in the CMS+fluoxetine or the control groups over the 4-month period (*p*>0.05).

**Fig 4:**
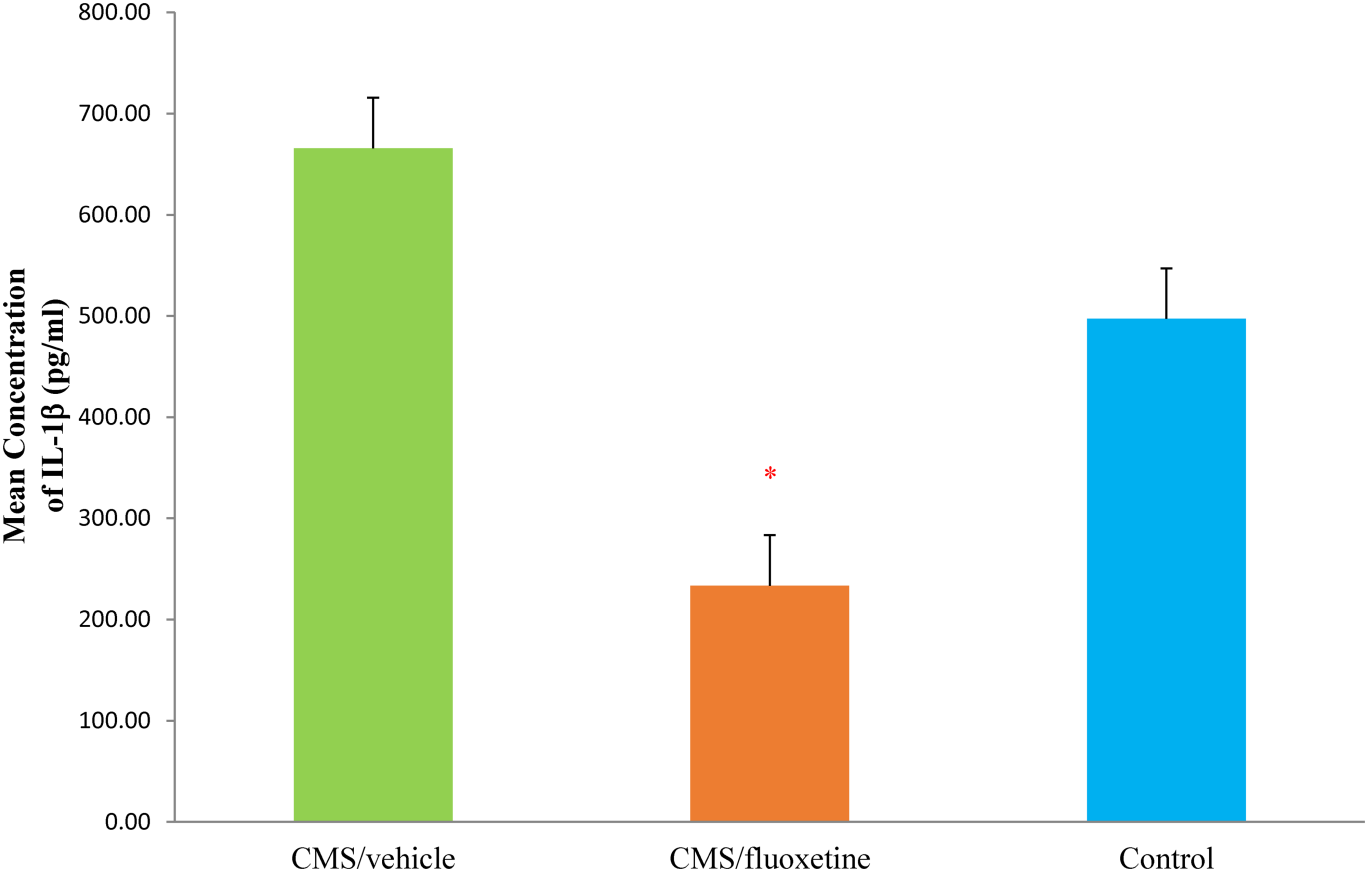
The effects of administration of fluoxetine and vehicle on IL-1β levels (day 120) in the brains of rats subjected to an animal model of chronic mild stress. Bars represent a mean+standard error. Control rats were not subjected to any stress or treatment. *p<0.05 *vs*. CMS+vehicle group according to Kruskal-Wallis test followed by Mann-Whitney U test.

In Table 2, the levels of four inflammatory cytokines (IL-1β, IL-17, IL-6, and TNF-α) in the left hemisphere were compared among the CMS+fluoxetine, theCMS+vehicle and the control groups by one-way ANOVA or Kruskal-Wallis test based on the normality of distribution in each group. One-way ANOVA showed that there was no significant difference in the levels of IL-6 (F = 3.241, df = 2, *p* = 0.076), IL-17 (F = 0.662, df = 2, *p* = 0.526) and TNF-α (F = 1.186, df = 2, *p* = 0.325) among the three study groups. However, Kruskal-Wallis test revealed a significant difference in the central levels of IL-1β (χ^2^ = 11.526, df = 2, *p* = 0.003). As shown in Fig 4, mean IL-1β level in the left hemisphere was significantly lower (*p*<0.05) in the CMS+fluoxetine group (233.3±20.07) pg/ml as compared to the CMS+vehicle group (665.75±150.32) pg/ml. There was a trend of lower central IL-1β level in the control group (497.2±137.33) pg/ml compared with the CMS+vehicle group (665.75±150.32) pg/ml, but this did not achieve statistical significance (*p*>0.05).

**Table 2:** Comparison of mean levels of inflammatory cytokines in the brain on day 120.

Cytokines	Groups	Day 120 (mean±SEM)	Test statistical value
**IL-1β**^#^	CMS+Fluoxetine (n = 9)	233.30±20.07	χ² = 11.526, df = 2, *p* = 0.003
	CMS+Vehicle (n = 9)	665.75±150.32	
	Control (n = 6)	497.20±137.33	
**IL-6**	CMS+Fluoxetine (n = 9)	6741.23±846.84	F = 3.241, df = 2, *p* = 0.076
	CMS+Vehicle (n = 9)	8094.00±1384.69	
	Control (n = 6)	5296.60±106.82	
**IL-17**	CMS+Fluoxetine (n = 9)	183.16±19.92	F = 0.662, df = 2, *p* = 0.526
	CMS+Vehicle (n = 9)	227.08±33.79	
	Control (n = 6)	231.59±51.87	
**TNF-α**	CMS+Fluoxetine (n = 9)	57.70±13.76	F = 1.186, df = 2, *p* = 0.325
	CMS+Vehicle (n = 9)	67.04±13.53	
	Control (n = 6)	31.29±19.93	

CMS = chronic mild stress; IL = interleukin; SEM = standard error; TNF = tumour necrosis factor. Data analysis was performed using one-way analysis of variance (ANOVA) and

^**#**^Kruskal-Wallis test.

## Discussion

In the present study of a 4-month (equal to 12 years of human) rat model of CMS, the impact of depression and chronic administration of fluoxetine on the peripheral and central levels of inflammatory cytokines was dynamically evaluated at baseline, day 60, day 90 and day 120. Our results showed that chronic depression significantly elevated the levels of IL-1β on day 60 onwards and the secretion of TNF-α and IL-17 on day 120 in rats undergoing chronic stress. A similar trend was observed with IL-6 levels in the CMS+vehicle group, but the results cannot be statistically proven from this investigation. Chronic administration of fluoxetine, a SSRI, effectively alleviated depressive symptoms including reducing a prohedonistic effect as shown by increased sucrose preference and enhancing vigorous activity as indicated by lowered immobility time. Furthermore, fluoxetine normalized the elevated production of inflammatory cytokines in plasma and brain during CMS, especially in reducing IL-1β level on day 60 and day 120. It can, therefore, be reckoned that IL-1β is the chief mediator of inflammatory cytokines in chronic stress response; TNF-α, IL-17 and IL-6 play a role in the inflammatory mechanism of depression as well.

Rats in the CMS+fluoxetine group had significantly higher sucrose preference than animals in the CMS+vehicle group who showed decreased percentage of sucrose preference upon the experience of CMS, indicating that fluoxetine has the capability of alleviating the anhedonic effect induced by depression. This finding is consistent with previous reports of fluoxetine effectively reversing anhedonia based on results derived from non-prolonged CMS models [34, 35]. A decrease in sensitivity to rewards in the CMS+vehicle group may be related to loss of body weight in rats, while the increase in sensitivity to rewards in the CMS+fluoxetine group may reflect an increase in body weight gain [36, 37]. In this study, the confounding of weight related effect on sucrose preference can be easily excluded because our results indicate a significant body weight gain in all the 3 groups, possibly explained by inactiveness of rats towards ageing. The forced swim test was conducted to complement sucrose preference and body weight results. In line with other studies, we found shorter immobility time in the CMS+fluoxetine group as compared to the CMS+vehicle group. Dang et al. [29] reported that the immobility time in CMS rats treated with fluoxetine or ginseng total saponins was significantly shorter than in rats subject to chronic stress and distilled water. The prolonged immobility time in the CMS+vehicle group may be a reflection of *arrested flight* and *entrapment* [38]. Forced swim test examined the effectiveness of fluoxetine in alleviating depressive symptoms via psychomotor activity.

Inflammatory cytokines such as IL-1β are proposed to play a critical role in mediating the development of anhedonic depressive-like symptoms. In mice with deletion of type I IL-1 receptor, 5-week CMS experience resulted in no decrease of sucrose preference, social exploration or neurogenesis at all when compared to wild-type mice [39]. Hence, this indicates that IL-1β is essential in depression in terms of the development of depressive symptomology and neurogenesis impairment. Fluoxetine is a SSRI antidepressant that counteracts depressive symptoms by inhibiting the reuptake of serotonin and thus, augments serotonin concentration [40]. The relatively high extracellular serotonin levels can inhibit the secretion of cytokines [41]. The present study found that IL-1β production in the periphery and brain was statistically lower in the CMS+fluoxetine group as compared to the CMS+vehicle group. Fluoxetine is effective in reducing IL-1β production over a 4-month period, with a significant decrease on day 60 and day 120. These results are in agreement with previous research that showed fluoxetine to be effective in reducing IL-1β serum level in depressed patients after 6 weeks of treatment [42]. The observations in this study extend the findings from the previous study by showing the effectiveness of long-term fluoxetine treatment in attenuating the high IL-1β production *in vivo* in a rat model of depression. However, the underlying mechanisms of the impact of fluoxetine on IL-1β reduction have been far from fully elucidated yet. In respect with this, a few hypotheses have been proposed that could possibly explain the role of fluoxetine in alleviating depressive symptoms via the inhibition of IL-1β production. Firstly, IL-1β can deplete tryptophan by reducing food intake via the induction of nuclear factor-*κ*B (NF-*κ*B) in depressed patients [43]. Secondly, high IL-1β level can enhance the activity of the enzyme indolamine 2,3-dioxygenase (IDO) [44], which result in shifting the metabolism of tryptophan towards kynurenine instead of serotonin synthesis [43]. The role of fluoxetine in inhibiting IL-1β production may shunt the metabolism of tryptophan more towards serotonin synthesis, thereby alleviating depressive symptoms [43]. Future studies are required to further investigate the exact mechanism of the impact of fluoxetine on IL-1β.

There was a trend of lower peripheral and central levels of IL-6, IL-17, and TNF-α in the CMS+fluoxetine group compared to the CMS+vehicle group, but the difference did not achieve statistical significant, possibly due to type II error as a result of the relatively small sample size. The findings on the effect of fluoxetine on inflammatory cytokine levels in the present study are partly in line with published data. Roumestan et al. [45] found that fluoxetine and desipramine inhibit the release of TNF-α in rats treated by LPS which is known to induce depressive symptoms. IL-17 is solely secreted by Th17 cells, whereby, the measurement of IL-17 in plasma or brain may not reflect its real concentration and function [46]. The role of IL-6 in depression is controversial. Results derived fromIL-6 knockout mice showed that IL-6 contributed to a slight augmentation of adrenocorticotropic hormone (ACTH) and glucocorticoids [47]. Furthermore, unlike IL-1β which induced elevations in norepinephrine catabolite, 3-methoxy, 4-hydroxyphenylethyleneglycol (MHPG), IL-6 administration in rats did not induce noradrenergic activation [47].

There are limitations in this pilot study. Firstly, the relatively small number of rats in the CMS+fluoxetine group, the CMS+vehicle group, and the control group may possibly cause the lack of statistical significance in the serum and brain levels of IL-17, IL-6 and TNF-α. Secondly, we tried our best to reduce the sufferings of rats during the CMS procedure, which may perhaps lead to the insufficient severity of CMS. Thirdly, although fluoxetine reverses depressive symptoms and is often administered to rats/mice under CMS or other animal depressive models [48–50], it remains a major limitation that this study did not include a fluoxetine treatment group without establishment of CMS model. Therefore, the findings from the present study warrant replication in future studies.

## Conclusions

In conclusion, the present study demonstrates that chronic depression results in high pro-inflammatory cytokine production and 4-month consecutive administration of fluoxetine could alleviate depressive symptoms, reduce anhedonic effect, as well as reverse the elevated secretion of inflammatory cytokines in plasma and brain during CMS since day 60 onwards. Furthermore, these findings from this study demonstrate a complex relationship between fluoxetine and its effect on levels of different pro-inflammatory cytokines in treating depression, whereby it has a greater ability to significantly reduce plasma and brain IL-1β levels in a rat model of CMS.
